# Cardiac magnetic resonance imaging in pediatric patients ≤ 18 years with suspected arrhythmogenic right ventricular cardiomyopathy (ARVC): a correlation to genetics

**DOI:** 10.1186/1532-429X-17-S1-P269

**Published:** 2015-02-03

**Authors:** Wieland Staab, Peter Lauerer, Martin Fasshauer, Ulrich J Krause, Jan M Sohns, Andreas Schuster, Christina Unterberg-Buchwald, Thomas Paul, Joachim Lotz, Michael Steinmetz

**Affiliations:** Pediatric Cardiology and Intensive Care, University Medical Center Goettingen, Goettingen, Germany; Diagnostic and Interventional Radiology, University Medical Center Goettingen, Goettingen, Germany; Cardiology and Pneumology, University Medical Center, Goettingen, Germany; German Center for Cardiovascular Research (DZHK), Goettingen, Germany

## Background

Arrhythmogenic right ventricular cardiomyopathy (ARVC) is associated with increased risk of sudden cardiac death. It is a progressive disease that can already manifest itself during childhood and adolescence. Since little is known about the early stage in pediatric patients, this study sought to determine the influence of right and left ventricular findings from cardiac magnetic resonance imaging (CMR) in pediatric patients ≤ 18 years with suspected ARVC in conjunction with positive genetic testing.

## Methods

In a consecutive series between September 2010 and December 2013 (38 months), 79 (14.0 ± 3.9 years, 46 male) young patients ≤ 18 underwent contrast-enhanced magnetic resonance imaging (CMR, 1.5 T Siemens Symphony) and genetic analysis for evaluation of clinically suspected ARVC. CMR parameters were derived from standard SSFP sequences, using comercially available segmentation software (QMass, Medis, Leiden, The Netherlands). CMR parameters were evaluated for predictive values, specificity and senstivity with regard to positive genetic test results.

## Results

12 patients revealed disease defining mutations in either the PKP2 or DSP gene. On CMR, 5 patients showed major criteria due to a combination of moderate to severe RV dysfunction and dilation as well as regional akinesia. Applying the revised Task Force Criteria (rTFC), 6 patients showed minor abnormalities such as mild RV dilatation, dys-synchronus RV contraction or regional akinesia. Overall 11 out of 12 (92%) patients with positive genetic characteristics were found to have major or minor abnormalities applying the rTFC. Positive predictive value (PPV) was 100%, negative predictive value (NPV) was 93%, sensitivity was 93% and specificity was 100%. Mean RV EDVI/BSA was 80 ± 16 ml^2^ and mean RV EF was 51 ± 8 % in the whole study population. A subgroup analysis revealed a significantly (p = 0.01) decreased mean RV EF of 36 ± 9 % and an increased RV EDVI/BSA of 101 ± 10 ml/m^2^ in 11 patients with major or minor abnormalities according to the rTFC.

## Conclusions

This is the first study applying the revised Task Force Criteria (rTFC) for the detection of ARVC to young patient's ≤ 18. In the current study, CMR revealed 11 out of 12 patient`s (major and minor rTFC) with positive findings in genetics with perfect positive predictive value and specificity. In conclusion, CMR is a valuable tool helping to identify ARVC already in pediatric patients, in order to initiate early prevention and treatement.

